# Honey and Its Role in Relieving Multiple Facets of Atherosclerosis

**DOI:** 10.3390/nu11010167

**Published:** 2019-01-14

**Authors:** Huong Thi Lan Nguyen, Naksit Panyoyai, Stefan Kasapis, Edwin Pang, Nitin Mantri

**Affiliations:** 1 The Pangenomics Lab, School of Science, RMIT University, Melbourne 3083, Australia; ntlanhuong77@gmail.com (H.T.L.N.); stefan.kasapis@rmit.edu.au (S.K.); eddie.pang@rmit.edu.au (E.P.); 2 Department of ScienceVietnam Institute of Agricultural Engineering and Postharvest Technology, Hanoi 10000, Vietnam; 3 Faculty of Agricultural Technology, Rajabhat Chiang Mai University, Chiang Mai 50300, Thailand; naksit_pan@cmru.ac.th

**Keywords:** Honey, composition, antioxidants, atherosclerosis, inflammation, oxidative stress, cholesterol

## Abstract

Honey, a natural sweetener has been used universally as a complete food and in complementary medicine since early antiquity. Honey contains over 180 substances, including sugars mainly fructose and glucose, water and a plethora of minor constituents such as vitamins, minerals and phytochemicals. The chemical composition of honey varies depending on floral origin, environment and geographical conditions. The sugar components dominate honey composition and they are accountable for sensory and physicochemical properties in food industry. Although present in small quantities, non-sugar components are the major contributors to the health benefits of honey. Our review summarizes and discusses composition of honey, its protective effects and possible action modes on risk factors of atherosclerosis.

## 1. Introduction

**Figure 1:**
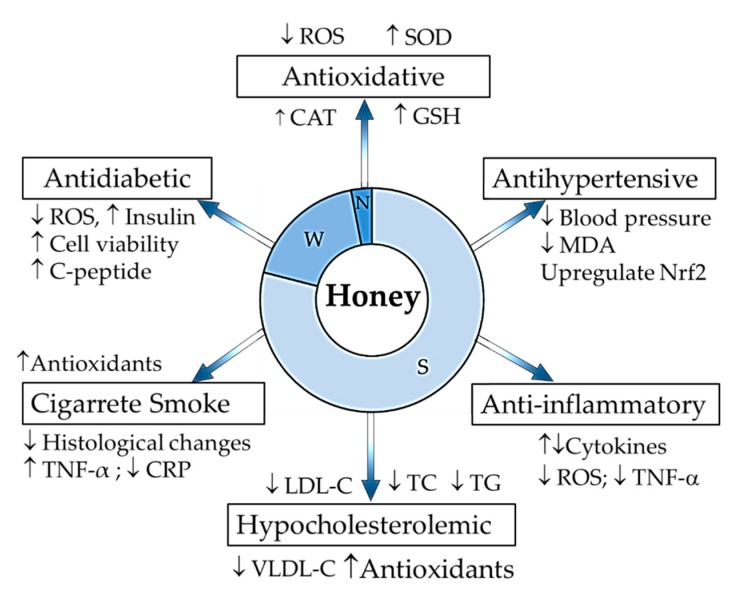
Summary of honey composition and its protective effects against risks in the pathogenesis of atherosclerosis. S: sugar components, W: moisture content, N: non-sugar components, ↓: decrease; ↑: increase; ROS: reactive oxygen species; SOD: superoxide dismutase; CAT: catalase; GSH: glutathione; MDA: malondialdehyde; Nrf2: nuclear factor erythroid 2-related factor 2; TNF-α: tumour necrosis factor alpha; LDL-C: low density lipoprotein cholesterol, TC: total cholesterol, TG: triglycerides, VLDL-C: very low density lipoprotein cholesterol; CRP: C-reactive protein.

Atherosclerosis is a chronic disease occurring in the inner lining of arterial walls due to the progressive plaque formation [1]. Multiple risk factors are implicated in the pathogenesis of atherosclerosis, including oxidative stress, inflammatory responses, hypercholesterolemia, hypertension, diabetes and cigarette smoking [2,3] (Figure 1). The factors are interrelated and their interactions may intensify the chronic disease [4]. Different strategies developed to relieve the risk factors covering gene therapy, synthetic antioxidants, vitamins and drugs, but atherosclerosis is still a leading cause of death worldwide [1].

Dietary antioxidants have attracted great attention as one of the most favourable options to combat the risk factors. Accumulating evidence indicates plant-originated antioxidant products are far more effective than synthetic counterparts in protecting and/or strengthening the endogenous defence and repairing mechanisms [5,6,7]. Among those, honey has been reported to exhibit a broad range of beneficial effects [8,9,10,11]. Honey has been reported as “a rediscovered remedy” and “a source of dietary antioxidants” [12,13,14,15].

Honey is a natural sweetener, contains mainly monosaccharaides (up to 80%), disaccharides (3–5%), water (17–20%) and a wide range of minor constituents such as vitamins, minerals, proteins, amino acids, enzymes and phytochemicals [16,17]. Its composition varies depending on botanical and geographical origin, as well as environmental conditions. The sugar components determine the energy value and its physicochemical properties which are critical for technological functions of honey [17,18,19]. Phytochemicals, mainly phenolic acids and flavonoids, are present in smaller quantities but they strongly determine the unique flavour, appearance and bioactivities of honey [17]. Phenolic compounds are known to offer complementary and overlapping modes of action through antioxidant activity, antibacterial and antiviral activities, modulating detoxification enzymes, stimulating the immune system, reducing platelet aggregation, modulating cholesterol synthesis and reducing blood pressure among the others [4,20,21]. Thus, their presence in the composition attributes to the relevant health benefits of honey [22]. Numerous studies have examined the phenolic profiles in honey and reported a high correlation of phenolic content with antioxidant capacity of honey [23,24].

Several excellent reviews have been dedicated to the characterization of honey composition and myriad of health benefits [15,17,25,26,27,28,29]. Our review summarizes and discusses: (i) the composition of honey and its key standards to ascertain its uniqueness, as why honey, mostly a sugar solution, elicits numerous health benefits, whereas table sugars are considered to have the reverse effect on health and contribute to growing epidemic of chronic illnesses; (ii) role of honey in relieving the multifaceted dimensions of atherosclerosis with possible action modes. Literature searches from Google Scholar, PubMed, ProQuest, Excerpta Medica dataBASE (EMBASE), ScienceDirect, Cumulative Index to Nursing and Allied Health Literature (CINAHL) and Scopus databases were performed and the keywords including “honey; antioxidant; composition; atherosclerosis, inflammation; oxidative stress; cholesterol” were entered for the reference selection.

## 2. Honey Composition and Antioxidant Activity

### 2.1. Honey Composition

Honey consists of over 180 components, including sugars, water and non-sugar components (Table 1) [30]. The sugar components in honey are mainly monosaccharides, particularly fructose (to 40%) and glucose (35.0%) in some honey types from Asia, Europe and Turkey, followed by a small quantity of disaccharides and higher sugars (<10%) [17]. Fructose and glucose in honey are derived from the chemical conversion of disaccharides in floral nectar by bee-secreted enzymes, where fructose is the highest proportion of any sugars in almost every honey type [15]. Sugars determine the physicochemical properties of honey such as viscosity, crystallization, thermal and rheological behaviour [19]. Sugars in honey provide an energy value of 300 kcal/100 gram honey, which is equivalent to 15% of recommended daily intake of energy [30]. Significantly, fructose contributes the highest proportion in almost every honey types (up to 45.0%) and it is a sweetest sugar among the natural sugars [15]. However, fructose has a lower glycaemic index (GI), compared to sucrose and glucose (GI at 15, 65 and 100, respectively) [31,32,33]. Since carbohydrate-containing foods are rated according to their GI, where low GI foods are absorbed more slowly from the gastrointestinal tract, fructose-rich honey varieties may be considered as a beneficial alternative to high GI sweeteners in management of diabetes and cardiovascular diseases [30,34].

The non-sugar components are at minor quantities, but they define a particular type of honey and bioactives, depending on the level of vitamins, minerals, antibiotic-rich inhibine, carotenoids, free amino acids, enzymes, proteins, Maillard reaction products and phenolic compounds present in honey composition [9,30]. Enzymes including invertase (saccharase), diastase (amylase), glucose oxidase and catalase play a critical role in honey formation. Particularly, invertase converts sucrose into monosaccharides, glucose oxidase catalyses hydrogen peroxide formation and catalase (CAT) supports the oxygen and water formation from hydrogen peroxide.

Interestingly, during nectar and pollen forage, honey bees transform phytochemicals from floral nectars of host plants into honey. The diversity of secondary metabolites in plants attributes to the variance phytochemical profiles in honey composition [35]. Phytochemicals in honey are mainly phenolic acids, flavonoids and their derivatives. Phenolic acids (e.g. caffeic, chlorogenic, coumaric, ellagic, ferulic, gallic, homogentisic, phenyllactic, protocatechuic, syringic and vanillic acids) comprise hydroxybenzoic and hydroxycinnamic acids. Hydroxybenzoic acids exert antioxidant capacity (AOC) based on the positions of OH groups in the aromatic ring, with gallic acid (3, 4, 5-trihydrozybenzoic acid) as the most effective antioxidant in this group [36]. Hydroxycinnamic acids present greater free radical scavenging ability because of the unsaturated chain bonded to the carboxyl group, imparting stability to the phenoxyl radical group. Hydroxycinnamic acids offer multiple hydroxyl groups to combat free radicals. In addition, the electron donor groups present in the benzene ring provide a greater number of resonant structures and increase the stability of the acrylic radicals in cinnamic acids [36,37].

**Figure 2:**
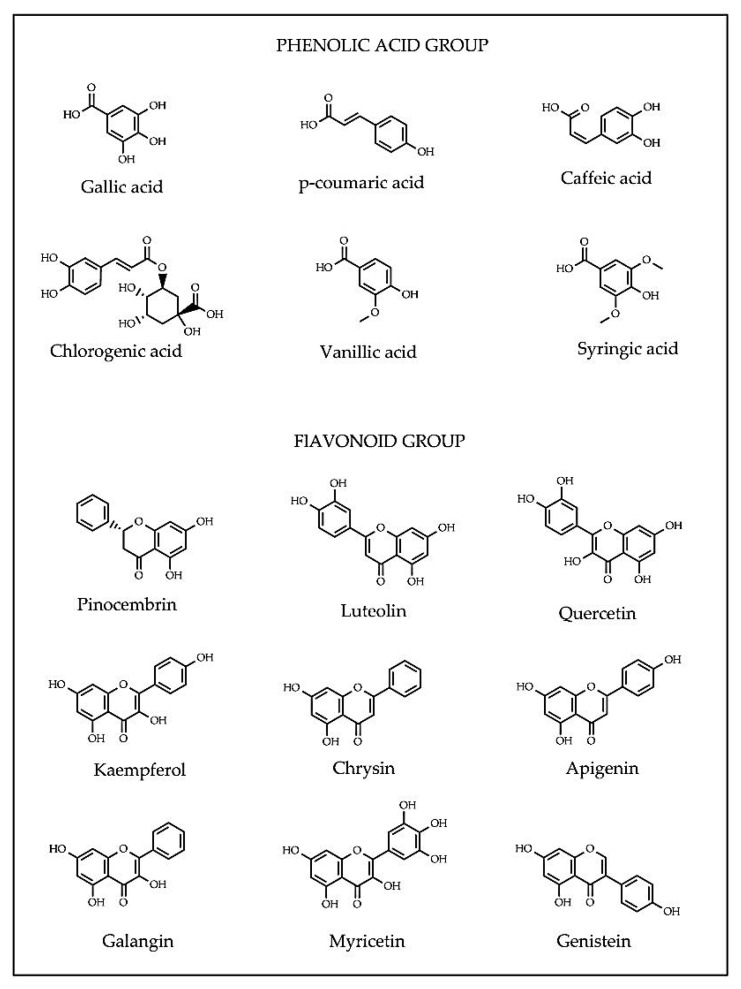
Common phenolic acid and flavonoid compounds identified in honey.

Flavonoids (apigenin, chrysin, galangin, hesperetin, kaempferol, luteolin, myricetin and quercetin) consist of two aromatic rings A and B, joined by a 3-carbon link, usually in the form of a heterocyclic ring C [36]. Variations in the ring C result in different flavonoid classes, including flavonols, flavones, flavanones, flavanols, isoflavones, flavanonols and anthocyanidins. Substitutions in rings A and B generate diverse compounds in each flavonoid class [38]. Depending on the molecular structures, phenolic compounds exert antioxidant capacity (AOC) in different action modes such as metal chelators, free-radical scavengers or gene modulators of enzymatic and non-enzymatic systems regulating cellular redox balance [39]. The presence of a specific phytochemical or combination thereof in honey may potentially serve as a marker for geographical and botanical origin of honey [40,41]. For examples, methylglyoxal is in manuka honey, hesperetin in citrus honey, quercetin in sunflower honey and luteolin in lavender honey [26,41,42,43]. The structures of common phenolic compounds in honey are presented in Figure 2.

During pollen and nectar forage, bees are exposed to the vegetation, soil, climate and water conditions located approximately within seven km^2^ in the vicinity of their hives [44]. The presence or deficiency of a particular element from the environment may be noticeable in the honey. Thus, the composition profile of honey not only reflects the quality and origin, it is also a bio-indicator of the environment [45].

To sum up, honey composition is complex and variable depending on its botanical and geographical origin. Each constituent has its nutritional, biological and technological functions. They synergistically contribute to the overall utility of honey, making honey unique and superior to other natural sweeteners in providing energy and health benefits.

### 2.2. Key Compositional Standards

The variations in honey’s composition, bee species, seasonal and storage conditions highlight the need for the quality standardization of different honey types. Key compositional criteria have been specified as common quality norms for commercial honey in both European Directive and in the Codex Alimentarius standard [46,47] (Table 2).

Moisture content (≤20%, w/w) is an important norm for honey, because high moisture content increases the value of water activity and promotes yeast growth leading to fermentation during storage. Exclusively, the osmotolerant yeasts such *Saccharomyces* spp. can grow in a low water activity value at 0.61 using a large amount of glucose and fructose in honey to produce alcohol and carbon dioxide [48]. Honey’s moisture content depends on the environmental, processing conditions during the harvesting period and storage.

Reducing sugars (glucose and fructose) and non-reducing sugars (sucrose, maltose) are physical attributes of honey, in particular crystallization process during storage. The amount of sugars in Australian honey is detailed for glucose (28.7–30.6%), fructose (32.8–36.0%), sucrose (1.1–2.2%) and maltose (1.1–2.2%) [49]. Saturated glucose is less soluble than fructose in honey, therefore glucose tends to form nuclei and expand to large crystals in aged honey, while fructose solution is stable in an amorphous state at ambient temperature. Crystallization of honey sugars depends on various factors including glucose concentration, fructose/glucose ratio (F/G) and water residue. Floral honey with a high concentration of glucose undergoes a relatively rapid crystallization compared to honeydew honey [50]. Honey possessing a F/G ratio > 1.3 does not crystallize during lengthy periods of storage, while at F/G < 1.1 its crystallization process occurs readily [51]. The crystallization process also depends on glucose and water content (G/W), whereby honey with G/W < 1.7 crystalizes slowly and with G/W > 2.0, the process is rapid and complete [52].

Electrical conductivity (EC) is a useful and reliable parameter for the determination of botanical origin of honey, since it is dependent and proportional to the content of minerals and organic acids in honey. These compounds are chemically ionizable, so they are capable of conducting electric current in solution [53]. EC is usually determined in a 20% honey solution (w/v) at 20 °C and expressed in milli or microsiemens per centimetre (mS cm^−1^, μS cm^−1^) [53]. Different types of honey showed varied EC values, particularly, honeydew honey (822–1213 μS cm^−1^), heather honey (815–1092 μS cm^−1^), citrus honey (124–262 μS cm^−1^) and rosemary honey (89–250 μS cm^−1^) [54,55]. Thyme honey types originated from Spain and Italy showed a similar EC range (288–559 μS cm^−1^) [54].

Free acidity, pH and water activity represent texture, stability and shelf-life of honey [56]. Free acidity originates from organic acids. Some studies reported its range of 16.1–34.1 meq/kg for Turkish honeys and around 40.0 meq/kg for Portuguese honeys [57,58]. High acidity is an indicator of sugar fermentation. Honey is usually acidic with a pH range of 3.2–4.5. The low pH and water activity (*a*_w_) values limit the growth of microorganisms.

Diastase activity and hydroxymethylfurfural (HMF) are markers denoting high temperatures and storage conditions. They are also dependent on the honey origin and climate region [59]. Diastase is susceptible to heating and storage factors, while HMF is almost devoid in fresh honey but present in processed and stored honey products. It is known that diastase activity is low and HMF value is high in heated honey [60,61]. Even though the enzyme activity is much more variable than the HFM value of a honey, they are both used for the selection of appropriate processing and packaging techniques among technological applications of natural honey [62].

Although honey colour is not listed amongst the standards, it attracts great attention because it is the first sign reflecting the physicochemical and biological properties of honey. Honey colour is also strongly depends on botanical origin, age, storage and processing conditions [18,26,56]. During storage, honey colour may become lighter as a result of the crystallization process, derived from the development of the white glucose crystals. Significantly, honey colour strongly correlates with the antioxidant potential of honey. Dark colour honey usually has higher ash and total phenolic content with resultant higher antioxidant capacities. For example, dark colour manuka honey (L* = 23.70, a* = 0.09; and b* = 0.15; colour intensity = 7296.7 mAU) showed a significantly stronger antioxidant power and higher phenolic content, compared to lighter colour honeys (L* = 24.90–27.31, a* = 1.42–2.10, b* = 2.66–3.59; colour intensity = 376.7–580.8 mAU) [63].

### 2.3. Antioxidant Capacity

The antioxidant capacity (AOC) of honey was reported to be the synergistic effect of mainly phenolic compounds along with other constituents in honey composition [28,64]. Considerable AOC values are well documented for a broad range of honey types from different botanical and geographical origins [12,18,43,58,65,66,67,68,69,70,71,72,73]. This notion was further supported by the fact that AOC value of honey is highly correlated to its phenolic content and colour intensity [23,24,64]. Interestingly, oxygen radical absorbance capacity (ORAC) value of honey was suggested to be equivalent to that of many fresh fruits and vegetables (3–17 µmol Trolox equivalent (TE)/g and 0.5–19 µmol TE/g fresh weight, respectively) [64].

The AOC of a sample is the basis for the quality comparisons, controls and the treatment of associated diseases [74]. The AOC of honey has been extensively examined using a number of popular chemical assays such as total phenolic content, free radical scavenging using 2,2-Diphenyl-1-picrylhydrazyl, trolox equivalent antioxidant capacity and ORAC among the others [23,71,75]. Findings from the assays, however are indicative of limits in either elucidating the total AOC due to the complexity of chemical components and the unique action mode of antioxidants [64] or potential bioactivity under physiological conditions [76]. Therefore, *in vitro, in vivo* and clinical evidence are crucial for further understanding not only AOC but also other biological activities of honey in providing health benefits, particularly attenuating the pathogenesis of atherosclerosis.

## 3. Honey in Relieving Multiple Facets of Atherosclerosis

### 3.1. Oxidative Damage

Oxidative stress occurs as a pathological condition due to an excessive generation of radical species over antioxidant defence system [77]. The radical species are represented by superoxide anion radical, hydroxyl, alkoxyl and lipid peroxyl radicals, nitric oxide and peroxynitrite [78]. They attack the cells, oxidize and damage proteins, lipids and deoxyribonucleic acids (DNA) randomly under stress conditions and excessive levels. Organisms have developed self-defence mechanisms towards neutralizing free radicals including repairing, physical defence and antioxidant systems. Enzymatic antioxidants are represented by superoxide dismutase (SOD), glutathione peroxidase (GPx) and catalase (CAT). Non-enzymatic antioxidants include ascorbic acid, α-tocopherol, glutathione (GSH), carotenoids, flavonoids and other antioxidants. The balance between the defence systems and free radical species generation is critical for their vitality [4]. The honey’s effects on oxidative stress have been the focus of several studies (Table 3). The mechanisms through which honey exerts the protection against oxidative damage resides in (i) antioxidant enzymes in its composition (such as catalase), (ii) phenolic compounds which chelate mental elements, trap or scavenge free radical species and induce cellular enzymatic and non-enzymatic antioxidant systems [24,26,39].

It was indicated that honey significantly inhibits the serum LDL oxidation compared to the sugar analogue and its ORAC values are correlated to its inhibitory effects against LDL oxidation [79]. In another study on the effects of five different honey beverages on human serum, the authors reported serum AOC in the ORAC assay increased by 7% (*p* < 0.05) after intake of buckwheat honey blended beverages (160 g/L), even though values of the serum lipoprotein oxidation and its by-product obtained from thiobarbituric acid reactive substances assay were insignificantly changed. However, this preliminary evidence potentially facilitates the ground works for long-term and epidemiological studies of the health benefits from consumption of honey-blended beverages [80].

The findings were supported by studies which reported the inhibitory effects of honey on lipoprotein oxidation of homogenates from rat liver, brain, lung, kidneys. Particularly, honey decreased the concentration of lipid peroxidation products namely H_2_O_2_ and malondialdehyde (MDA). This protection is related to the antioxidant activity of honey, which is comparable to those of melatonin and vitamin E [81]. In a later study, Alvarez Suarez et al. [82] found that vine honey displayed the highest capability in scavenging 2,2-Diphenyl-1-picrylhydrazyl, hydroxyl and superoxide radicals among the tested honey group and this honey elicited the remarkable inhibitory capacity against lipid oxidation in rat liver homogenate.

Endothelial cells play an important role in homeostasis, immune, inflammation, cell adhesion, thrombosis and fibrinolysis regulation, thus endothelial dysfunction initiates atherosclerotic progression [91,92]. Significantly, honey has been shown to enhance endothelial function through quenching lipophilic cumoxyl and cumoperoxyl radicals, suppressing cell damage, inhibiting cell membrane oxidation and decreasing reactive oxygen species (ROS) generation and GSH recovery in EA.hy926 endothelial cells [87].

Although human red blood cells (RBCs) are not directly related to atherosclerosis, their alterations may enhance the severity of atherosclerosis. RBCs are sensitive to oxidative damages due to the structural and functional characteristics, thus lipid oxidation of erythrocyte membrane causes the cell death or erythrocyte haemolysis. Honey flavonoids have been reported to prevent the peroxidation process of the lipid membrane, intracellular GSH depletion and SOD decline in RBCs, thus protect the cells from oxidative haemolysis and reduce extracellular ferricyanide [82,83,84]. Studies have suggested flavonoids localize in the membrane bilayer and form specific bindings to lipids and proteins in RBC membranes. As a consequence of this process, the membrane is protected from the peroxidation and strengthened against the stress factors [85,93]. Alternatively, flavonoids such as quercetin may be incorporated into RBCs to exert antioxidant effect on RBC membranes [83,86].

Gelam honey has also been evidenced to reduce MDA level, a product of peroxidation process and protect DNA oxidative damage in both the young and aged rats. The honey increased the activity of antioxidant enzymes namely erythrocyte CAT and cardiac SOD in young group, while increasing the activity of only cardiac CAT in both of the young and aged groups. The authors suggested that the reduced oxidative damage in honey-fed rats was related to the elevation of antioxidant enzyme activity under the effect of Gelam honey [88]. Another investigation revealed that honey promoted higher plasma tocopherol content, tocopherol/triglyceride level but lower plasma NOx levels and a reduced susceptibility of heart towards lipid oxidation in the honey-fed rats compared to the control [89]. The findings are consistent with results from a previous study in human plasma, where honey consumption increased plasma total phenolic content (*p* < 0.05), antioxidant and reducing capacities (*p* < 0.05) [90]. Therefore, honey substitution as a sweetener provides health benefits through the enhancement of the antioxidant defences.

The cardio-protective effect of honey has been further demonstrated in urethane-anesthetized rats administered with epinephrine whereby honey pre-treatment (5 g/kg) for one hour was found to reduce the epinephrine-induced incidence in anesthetized normal rats while honey post-treatment significantly prevented the incidence in anesthetized stressed rats. The studies suggested that the pronounced antioxidant components in honey contributed to the protection of cardiovascular system [94].

Taken together, the studies reported antioxidant capacity of honey from different origins in different models and the mechanisms through which honey exerts its antioxidant activity resides in (i) antioxidant enzymes in its composition (such as catalase), (ii) the high content of phenolic compounds which chelates mental elements, traps or scavenges free radical species and induce cellular enzymatic and non-enzymatic antioxidant systems [24,26,39]. It should be taken into considerations that the total antioxidant capacity of honey resulted from the synergistic interaction of different compounds, including phenolics, peptides, organic acids, enzymes, Maillard reaction products and other minor components. However, due to a loss of up to 40% in total phenol content and total antioxidant activity during the fractionation process [87,95], the overall effects of honey on oxidative damage obtained from the tested fractions could be underestimated. In addition, some studies evaluated the effects of honey on lipid peroxidation of tissue homogenates which may contain a wide range of compounds (proteins, intracellular lipids and others) interfering with the test specificity. Thus, it is suggested that measurement of generated lipid hydroperoxide concentration is more indicative and specific for the evaluation [82]. To date, the investigations on honey’s antioxidant effects *in vitro* and *in vivo* focus on the aqueous portion of the blood (plasma) where honey antioxidants dissolve, it is suggested that the future studies should progress to evaluate the honey’s effects on the lipid components of the human body [90]. Given the limitations, the findings are supportive to the hypothesis that honey could play a role in protecting biological systems from oxidative damage.

### 3.2. Inflammatory Responses

Inflammation reflects a pathophysiological response of tissues characterized by signs of pain, heat, redness and swelling [96], however, prolonged inflammation is the cause of several chronic diseases such as diabetes, dyslipidaemia, hypertension, cardiovascular, obesity and pulmonary conditions. Under inflammatory conditions, mitogen-activated protein kinase (MAPK) and nuclear factor kappa B (NF-κB) pathways are activated, triggering several important proinflammatory markers including cyclooxygenase-2 (COX-2), lipoxygenase 2 (LOX-2), C-reactive protein (CRP), interleukins (IL-1, IL-6 and IL-10) and tumour necrosis factor alpha cytokine (TNF-α) [27]. Honey was found to modulate the inflammatory response in the pathogenesis of atherosclerosis through distinct inhibitory paths of (i) proinflammatory markers such as cytokines, COX-2, CRP and TNF-α [97,98,99,100] and (ii) ROS generation [101].

It was reported that the anti-inflammatory activity of honey is contributed by phenolic compounds and other minor constituents in its composition [101,102,103,104]. Kassim et al. detected a range of phenolic compounds, including chrysin, quercetin, ferulic acid, ellagic acid, hesperetin in Gelam honey. This honey reduced cytokine (TNF-α, IL 1β and IL 10) and NO levels but increased heme oxygenase-1 levels. Thus, the honey was recommended to be further investigated for treatment of different inflammatory diseases [100]. Some phenolic compounds have been individually examined for their anti-inflammatory activity. Chrysin was reported to suppress lipopolysaccharide-induced COX-2 in Raw 264.7 cells [97]. Luteolin was found to reduce intercellular adhesion molecule-1 and TNF-α and eradicate leukocyte infiltration in tissues [99]. Quercetin was demonstrated to reduce human CRP expression and also serum amyloid A and fibrinogen which are cardiovascular risk factors in mice [98].

The findings are supportive to a study on the anti-inflammatory effect of a natural honey type on bovine thrombin-induced oxidative burst in human neutrophils and rodent macrophages. It has been known that the accumulation of phagocytes, ROS production and thrombin activation occur at the sites of endothelial damage [101]. It was demonstrated that bovine thrombin-activated phagocytes produce ROS which might amplify the inflammatory responses at the site of atheromatous plaques. However, honey treatment suppressed the thrombin-induced ROS generation by the phagocytes. The findings suggested a beneficial role of honey in the pathology of atherosclerosis, particularly in ROS-induced LDL oxidation and cell signalling [101].

### 3.3. Hypercholesterolemia

Cholesterol is an indispensable molecule in growth and development of animal and human cells. It fulfils vital functions such a cell membrane component, a precursor for steroid hormones and bile acids and an activator in cell signalling pathways [105]. Cholesterol is combined with lipoproteins so that they are transported from one tissue to the others throughout the body. Lipoproteins are divided into high density lipoprotein (HDL), low density lipoprotein (LDL) and very low-density lipoprotein (VLDL), thus cholesterol (C) is classified accordingly into HDL-C (good cholesterol), LDL-C and VLDL (bad cholesterols) [106].

A high level of LDL-C is the main cause of plaque formation in blood vessels, which when occurred in coronary arteries, it results in blockages and heart attacks [7]. In addition, a marked elevation of lipid oxidation products and/or a reduction in plasma antioxidants promotes hypercholesterolemia [118]. Use of dietary antioxidants combined with physical exercises has been recommended as a premised lifestyle approach to control cardiovascular risks in general and cholesterol levels in particular [119].

Containing an abundant source of phenolic compounds [9,15,115], honey has been shown to improve lipid profile, particularly cholesterol levels (Table 4). The exact mechanism of honey in the improvement of this risk factor has not been clearly determined. However, phenolic compounds present in honey are reportedly associated with improvement of coronary vasodilation, prevention of blood clots and protection of LDL-cholesterol from oxidation [120]. Several natural phenolics have been reported to reduce cholesterol, including quercetin-3-β-D-glycoside, vanillin rich fraction and luteolin among the others. The phenolic compounds have been known to (i) decrease cholesterol level through the inhibition of 3-hydroxy-3-methylglutaryl co-enzyme A (HMG-CoA) reductase which is a crucial rate limiting enzyme in cholesterol biosynthesis, and/or (ii) modulate plasma LDL-C via the upregulation of LDL-receptor (LDLR) expression, of which LDLR is a cell surface glycoprotein important to the hepatic uptake and removal of plasma cholesterol [121,122,123,124]. It has been demonstrated that honey is a potential alternative for sucrose intake in individuals with poor glycaemic control and/or coronary heart disease. In a study, the long-term 52 week consumption of honey did not result in any differences in LDL-C, triglyceride (TG) or total cholesterol (TC) levels among the rat groups. However, honey diet revealed a significant increase in HDL-C levels (16% to 21%) in honey diet rats, compared to sucrose (*p* = 0.044) or sugar-free diet group (*p* = 0.006) [107].

High carbohydrate diets are connected to obesity and impaired adipose metabolism. The effects of honey on weight gain, adiposity and related biomarkers were evaluated in a study feeding rats with clover honey (honey diet group) and compared with sucrose (sucrose diet group) for 33 days. The authors found that the honey diet reduced body weight (*p* ≤ 0.05) and serum TGs concentrations (*p* ≤ 0.05) compared with the relevant sucrose diet. However, honey did not result in significant differences in serum HDL-C and TC [108]. In another study, honey significantly increased TG, HDL and VLDL levels and decreased plasma LDL and TC levels compared to the control group [110]. The findings are consistent with results from a recent study comparing the ameliorating effects of honey on hyperglycaemia and hyperlipidaemia in diabetic rats fed with honey for 3 weeks. The study found that use of honey (1.0 and 2.0 g/kg) increased HDL-C (*p* < 0.05) and reduced hyperglycaemia, TGs, VLDL-C, non-HDL-C, coronary and cardiovascular risk index (*p* < 0.05). However, honey at higher dose (3.0 g/kg) reduced only TGs and VLDL-C (*p* < 0.05) [109].

These results are comparable to findings from the examination of the effects of Gelam and Acacia honey on weight gain and obesity-related parameters using male Sparague-Dawley rats fed with high cholesterol diet (HCD) before treatments. The study reported a reduction in excess weight gain and adiposity index in honey group compared to control group. The honeys and the orlistat drug which elicited hepatotoxicity effects showed similar effects in significantly reduced levels of plasma glucose, triglycerides and cholesterol, obesity related parameters in rats. The authors suggested Gelam and Acacia honey are more effective than orlistat in obesity control through regulation of lipid metabolism [113]. The finding was supportive to the investigation on the renoprotective effect of Tualang honey on HCD fed rats. It was found that the TC and TG levels were markedly decreased in the honey group compared to the control at 7 days (*p* = 0.025 and 0.031, respectively). The honey group also was found to have considerably lower serum creatinine level than untreated group after 48 h (*p* = 0.018). This study indicated that the honey showed some degree of renoprotective effect biochemically [112]. Tualang honey was also examined by another research group using isoproterenol-injected rats. Isoproterenol can cause severe oxidative damage in the myocardium leading to infarct-like necrosis in the heart muscle when administered in large doses. It was reported that isoproterenol-induced rats exhibited a significant elevation of serum TC, TGs, cardiac marker enzymes (creatine kinase-MB, lactate dehydrogenase) and aspartate transaminase, cTnI and also a decrease in antioxidant enzymes. However, the oral administration of Tualang honey (3 g/kg) for 45 days prior to isoproterenol treatment modulated TG, recovered the antioxidants and the mentioned parameters in rats [111].

The effect of honey on lipid metabolism was further confirmed by Bezerra et al. [114]. In this study, honey from *Mimosa quadrivalvis* L. produced by the *Melipona subnitida* D. (jandaira) stingless bee was evaluated for its effectiveness on lipid parameters, an antioxidant status and intestinal health of dyslipidaemic rats (1 g/kg) for 35 days. It was found that the honey group demonstrated lower food consumption, increased glucose tolerance and SOD activity, decreased total cholesterol, LDL and aspartate aminotransferase hepatic enzyme. Honey also increased beneficial bacterial population (*Bifidobacterium* spp. and *Lactobacillus* spp.) and organic acid excretion detected in faeces of dyslipidaemic rats. Taken together, honey administration showed the positive effects on the modulation of metabolic disorders and lipid profile improvement in a dose- and time-dependent manner, however, the implications of these findings need to be clarified through further animal studies.

Human clinical studies have been conducted as an addition to the *in vitro* and *in vivo* studies to further understand the effect of honey on lipid profile. Waili [115] investigated the effect of honey consumption on diabetic and hyperlipidaemic subjects for 15 days. In healthy subjects, honey consumption was found to decrease TC (7%), LDL-C (1%), TGs (2%), C-reactive protein (7%), homocysteine (6%) and plasma glucose level (PGL) (6%), while HDL-C (2%) levels were elevated. However, in patients with high blood lipid profile, the effect of honey was more pronounced in reducing TC (8%), LDL-C (11%) and CRP (75%), while sugar analogues increased LDL-C levels [115].

The findings were further supported by an independent clinical study with 55 obese individuals divided into two groups, an experimental group (*n* = 38) that daily consumed 70 g of Iranian natural honey and a control group (*n* = 17) consumed 70 g of sucrose for 30 days. The authors found honey exhibited positive effects on cardiovascular (CDV) risk factors, homocysteine and CRP without side effects and significant weight increase. In details, honey decreased TC (3%), LDL-C (5.8%), TGs (11%), fasting blood glucose (4.2%) and CRP (3.2%), while it increased HDL-C (3.3%) in individuals with the normal CDV parameters. However, honey showed a more noticeable effect in the reduction of TGs (19%) in individuals with the abnormal parameters [116].

The effect of natural honey was explored in patients with type 2 diabetes for lipid variables and body weight. The patients were divided into honey group (*n* = 25) and non-honey group (control, *n* = 23) for 8 weeks. After baseline adjustment, the fasting blood glucose in the two groups were not significantly different, however, the honey group showed a significant reduction in body weight, TC, LDL-C and TGs and dramatic increase in HLD-C (high density lipoprotein cholesterol) levels [32].

The findings are congruent with a recent study by Whitfield et al. [117]. In this study, the formulation of Kanuka honey with cinnamon, chromium and magnesium was investigated for its effect on glycaemic control, weight and lipid profile in 12 patients with type 2 diabetes. Consumption of the 53.5 g honey blend for 40 days significantly increased the body weight and improved lipid parameters in the subjects. In addition, a tendency in the increase of HDL and reduction of systolic blood pressure was also observed. However, the formulation did not affect glucose metabolism or glycaemic control.

Recently Tul-Noor et al. [125] reviewed clinical studies and undertook meta-analysis to assess the effects of honey intake on lipid risk factors, compared to sugar analogues. The authors found 10 eligible trials with a total of 444 samples, median period of 5 weeks and an average honey dose of 70 grams/day. They reported that regular administration of honey results in a reduction in LDL-C (*p* = 0.02), fasting triglycerides (*p* < 0.001) and an increase in HDL-C level (*p* < 0.001). They also found evidence of substantial inter-study heterogeneity for LDL-C (*p* < 0.001) and non-significant heterogeneity for fasting triglycerides and HDL-C (*p* > 0.10). However, the overall quality of the evidence in the analysis was assessed as “low quality” for LDL-C, “moderate quality” for fasting triglycerides and “moderate quality” for HDL-C according to the consistency and precision of data and publication variance. The authors recommended that honey intake showed a beneficial effect on lipid profile including LDL-C, TGs and HDL-C in participants at different health backgrounds but trials need to be at larger, longer scale and higher quality [125].

Although human clinical studies on cholesterol-lowering effects of honey are scattered, limited in size and time course, overall these studies have indicated a consistent and promising effect of honey in improving overall lipid profile, particularly reduced LDL-C and TGs and increased HDL-C in the research objects. Natural honey contains mainly fructose and glucose components, however the studies showed its positive effects outweighed sugar analogues on lipid profile [115,116]. The results suggest a functional role attributed to the non-sugar components in honey composition. Plant phenolic antioxidants are reported to be effective in improving blood lipid profile, thus they are possibly the primary contributors to those positive effects of honey [122,124,126]. Nonetheless investigations on the underlying mechanism of honey on hypercholesterolemia have not yet provided strong evidence and need larger clinical studies to explore further the mechanism.

### 3.4. Hypertension

Hypertension is closely implicated in the pathogenesis of atherosclerosis. Recent studies which reported honey reduced systolic blood pressure and MDA levels in hypertensive rats [127] and alleviated the susceptibility of rat kidneys to oxidative damage through upregulating the expression of erythroid 2-related factor 2 (Nrf2), an important transcription factor regulating antioxidant defences in chronic renal failure or hypertensive rats [128]. The results have indicated that the protective effect of honey on hypertensive rats is mainly contributed by its antioxidant and anti-inflammatory activity.

### 3.5. Diabetes

Diabetes is implicated in inflammation, oxidation and glycation. Therefore, strong antioxidant agents potentially limit the pathogenesis of diabetes and the associated complications [129]. Gelam honey extract has been found to protect pancreatic hamster cells from hyperglycaemic conditions. Significantly, this honey decreased ROS production, glucose-induced lipid peroxidation, increased insulin content and the cell viability under hyperglycaemic conditions [130]. The findings were supported by an investigation of Jujube honey for its role in modulation of the main enzymes participating in glucose metabolism namely glucokinase and glucose 6-phosphatase in rats. Jujube honey was found to reduce MDA levels while improving the total AOC in diabetic rats (*p* < 0.05). It also decreased heat shock protein (HSP70) and glucose 6-phosphatase expressions, while increasing the glucokinase expression [131].

Moreover, a pilot study with 20 patients with type 1 diabetes and 10 healthy controls showed honey treatment reduced glycaemic index and the peak increment index in both patients (*p* < 0.001) and control (*p* < 0.05) groups compared to sucrose. In this study, honey significantly increased C-peptide level, compared to either glucose or sucrose in the control group. The results suggested honey may be used as a sugar substitute for patients with type 1 diabetes [132]. Collectively, the findings suggested potential effect of honey on diabetes management in animal models should be translated into larger clinical trials for type 1 diabetic patients.

### 3.6. Cigarette Smoking

The active or passive exposure to cigarette smoking is implicated in all stages of atherosclerosis and complicates cardiovascular events [133]. Tualang honey was examined for its protective effect on rats exposed to cigarette smoke. It was found that honey protected rat testis from oxidative stress caused by tobacco smoking. The honey decreased the histological changes and lipid peroxidation, but it increased the total antioxidant levels and recovered the activity of antioxidant enzymes, particularly glutathione peroxidase, SOD and catalase in the cigarette smoke-exposed rats [134].

The findings were further supported by a recent study which examined the effect of a 12-week honey administration on plasma inflammatory markers such as highly sensitive CRP, IL-6 and TNF-α among 32 non-smokers and 64 chronic smokers [135]. The study reported that TNF-α was significantly increased, but CRP expression was significantly reduced at post-intervention among smokers with honey group. These indicated that effects of honey on TNF-α and CRP are opposite, thus it raises the needs for further investigations on the inclusive effect of honey on inflammation among chronic smokers.

## 4. Adverse Effects of Honey

Despite the nutritional and medicinal values, honey is prone to microbial and non-microbial contaminations. Several microorganisms including bacteria, moulds, yeast from pollen, bee intestine, human, equipment, containers and dust may infect honey. However, honey has antimicrobial properties due to the synergistic contributions of saturated sugars (~80%), acidic pH, bee defensin 1, inhibines (hydrogen peroxide, flavonoids and the phenolic acids) and low water activity [136,137]. However, spore-forming bacteria can resist for over a year in honey at low temperature [138,139], particularly the *Clostridum botulinum* causing botulism poisoning was detected in many countries [138,140,141,142]. Thus, raw honey that was not sterilized or qualified should not be used for infants. It was also recommended that *Clostridia* spores need to be eliminated from honey using gamma irradiation, a sterilization process which is not interfered with antibacterial activity of honey [143].

In addition, honey may contaminate with traces of pesticides, herbicides, antibiotics or heavy metals due to the bee disease control and the exposure of honey bees to environment [139]. Honey also may contain poisonous compounds, particularly grayanotoxins found in mad honey which originates from *Andromeda* flowers [144]. Thus, honey needs to be subjected to quality analysis and labelling regulations. Moreover, honey production and processing have to comply with standard protocols and legislation to assure its safety.

## 5. Conclusions

Honey composition is a mixture of saturated sugar and non-sugar constituents, varying accordingly to the environment, botanical and geographical origin. Dominant sugars are fructose followed by glucose, so honey is a lower GI product compared to table sugars. The non-sugar constituents such as enzymes, amino acids, vitamins, minerals, phenolic compounds are at minor quantities, but they define health benefits of honey. Each of them has its own nutritional and functional value(s) and they work together to contribute to the biological and physicochemical properties of honey, making honey a unique sweetener.

Atherosclerosis is a damaging chronic disease globally. Interestingly, several studies have emphasized the role of honey in attenuating the aforementioned risks in the pathogenesis of atherosclerosis. The beneficial effects are mainly attributed to phenolic compounds in honey composition. The mechanisms through which honey elicit the protection are associated with scavenging radical species, suppressing lipid peroxidation, strengthening enzymatic and non-enzymatic antioxidant systems and stimulating/inhibiting proinflammatory markers. However, further research in particular clinical translations will progress to better management strategies of the chronic disease, with concomitant expanded applications of honey in food and pharmaceutical industries. In addition due to possible microbial and non-microbial contaminations, honey quality should be complied with safety regulations and international standards.

## Figures and Tables

**Table 1:** Chemical composition of honey per 100 g [30].

Proximates (g)		Minerals (mg)		Vitamins (mg)	
Fructose	38.2	Calcium	3–31	Ascorbic acid	2.2–2.5
Glucose	31.3	Potassium	40.0–3500.0	Thiamin	0.0–0.01
Sucrose	0.7	Copper	0.02–0.60	Riboflavin	0.01–0.02
Other disaccharides	5.0	Iron	0.03–4.00	Niacin	0.1–0.2
Water	17.1	Magnesium	0.7–13.0	Pantothenic acid	0.02–0.11
Organic acids	0.5	Manganese	0.02–2.0	Pyridoxine (B6)	0.01–0.32
Proteins, amino acids	0.3	Phosphorus	2.0–15.0		
		Sodium	1.6–17.0		
		Zinc	0.05–2.00		
		Se	0.001–0.003		

**Table 2:** Key compositional standards of blossom honey [46].

Criteria	Values
Moisture content (%)	≤20.0
Fructose and glucose (Sum, g/100 g)	≥60
Sucrose (g/100 g)	≤5.0
Water-insoluble content (g/100 g)	<0.1
Electrical conductivity (mS/cm)	≤0.8
Free acid (meq/kg)	≤50.0
Diastase activity (Schade scale)	≥8.0
Hydroxymethylfurfural (HMF, mg/kg)	≤40.0

**Table 3:** Effects of honey on oxidative stress.

Honey Type	Research Model	Main Findings on Honey Effects	Reference(s)
Local honey	Rat kidney, brain, liver and lung homogenates	↓ Lipid hydroperoxides and malondialdehyde (MDA) value	[81]
Christmas vine, Morning glory, black mangrove, linen vine singing bean honey	Rat liver homogenates	Highest radical scavenging capacity in linen vine honey ↓ Lipid peroxidation	[82]
Fireweed, tupelo, Hawaiian Christmas berry clover, acacia, buckwheat, soybean honey	Human blood serum	AOC is different among honeys, ↓ Lipoprotein oxidation (LPO) Correlation of ORAC value and LPO inhibition.	[64]
Acacia, coriander, sider and palm honey	Human LDL	High antioxidant activity in xanthine-xanthine oxidase system and LDL oxidation	[79]
Buckwheat honey	Human blood serum	↑ Serum antioxidant capacity	[80]
Multifloral honey	Human red blood cells (RBC)	↓ Lipid peroxidation	[83]
Multifloral honey	RBC	↓ Extracellular ferricyanide level	[84]
Christmas vine, linen vine honey	RBC	Protection of human erythrocyte membranes from oxidative damage ↑ Defence responses and ↑ cell functions	[82,85,86]
Native multifloral honey	Endothelial cell (EA.hy926)	Protection of EA.hy926 from hydrogen peroxide and peroxyl radical Synergistic effect of phenolic antioxidants in honey	[87]
Gelam honey	Rat blood sample	↑ Antioxidant enzyme activities	[88]
Multifloral honey	Rat plasma and heart tissue	↓ Hypertriglyceridemia and pro-oxidative effects ↑ Plasma α-tocopherol and α-tocopherol/triglycerides, ↓ plasma NOx, ↓ peroxidation	[89]
Buckwheat honey	Human blood plasma	↑ Plasma antioxidant activity, ↑ defences against oxidative stress	[90]

AOC: antioxidant capacity, ORAC: oxygen radical absorbance capacity, LPO: lipoprotein oxidation, LDL: low density lipoprotein, RBC: Human red blood cells, TG: triglycerides, NOx: nitrogen oxides.

**Table 4:** Effects of honey on lipid profile.

Honey Type	Research Model	Main Findings of Honey Effect	Reference(s)
Honeydew honey	Rat blood serum	Similar weight gain and body fat in honey and control group; ↓ HbA1c, ↑ HDL-C	[107]
Clover honey	Rat blood serum	↓ Weight gain and adiposity, ↓ TGs but ↑ non-HDL-C levels	[108]
Native honey	Rat blood samples	↓ glucose and lipids no deteriorated effects on hyperglycaemia and dyslipidaemia	[109]
Local honey	Rat blood serum	↑ Plasma TG, HDL-C and VLDL-C but ↓ plasma LDL-C and TC	[110]
Tualang honey	Rat heart tissue	↑ Antioxidant enzyme levels in heart tissue and ↓ lipoprotein oxidation (LPO)	[111]
Tualang honey	Rat blood serum, kidneys	↓ TC and TG compared to the control at 7 days; ↓ Serum creatinine level than no honey group after 48 h; No structural effect histologically in the HCD-fed rats	[112]
Gelam, Acacia honey	Rat blood serum, internal organs	↓ Excess weight gain and adiposity index; ↓ plasma glucose, TGs, TG and obesity at similar levels to orlistat drug group	[113]
Malícia honey	Rat blood serum, liver	↓ Food consumption, ↑ glucose tolerance and SOD activity; ↓ TC, LDL and AST levels; ↑ beneficial bacteria and organic acids; Colon and liver was protected	[114]
Natural local honey	Healthy, diabetic and hyperlipidaemic human subjects, blood samples	↓ Blood lipids, homocysteine and C-reactive protein (CRP) in normal and hyperlipidaemic subjects; ↓ plasma glucose elevation in diabetics	[115]
Natural honey	Human plasma	↓ TC (3.3%), LDL-C (4.3%), TGs (19%) and CRP (3.3%) in elevated variable subjects; No increased body weight in overweight or obese participants	[116]
Natural unprocessed honey	Type 2 diabetes human subjects, weight and blood samples	↓ Body weight, TC, LDL-C, TGs ↑ HDL-C and HbA1C levels	[32]
Kanuka honey, formulated with cinnamon, chromium and magnesium	Type 2 diabetes human subject, weight and blood samples	↓ Weight Improve blood lipid profile	[117]

HbA1c: Haemoglobin A1c, HDL-C: high density lipoprotein cholesterol, LDL-C: low density lipoprotein cholesterol, VLDL-C: very low density lipoprotein cholesterol, TC: total cholesterol, TGs: triglycerides, LPO: lipoprotein oxidation, HCD: high cholesterol diet, AST: aspartate aminotransferase, CRP: C-reactive protein.
